# Supra-pubic Lithotomy

**Published:** 1883-03

**Authors:** 


SUPRA-PUBIC LITHOTOMY. By Wm. Tod Helmuth, M. D., Professor of Surgery in New York Homœopathic Medical College. Bœricke & Tafel, New York and Philadelphia. 98 quarto pages, 8 lithographic plates. Price, $4.00.

As far as we know, this is the most carefully compiled, complete, best arranged, and most instructive monograph on the subject. The author has here presented a permanent contribution to surgery and deserves the thanks of the whole profession, due credit being given throughout the book to Dr. C. W. Dulles, of this city, for free use of materials gathered for his several articles, which were the first, in late times, to call the attention of the profession to the merits of this neglected operation. Chapter second, prepared by Dr. Rankin, gives the statistics, as near as it is possible to collate them, and represents a vast amount of useful labor. Dr. Helmuth's four cases are included—two recoveries and two deaths.

The general summary of cases since 1561, according to Rankin and Dulles, shows a total of five hundred and twenty-seven operations, with three hundred and fifty-eight recoveries, one hundred and thirty-five deaths, and thirty-four unknown results. Chapter third discusses the subject and gives plates illustrative of interesting experiments made by injecting the bladder, showing the relative position of the pubes of the reflected peritoneum and the amounts of fluid the bladder is capable of holding. Chapter four gives the particulars of eight cases by different operators, interesting in their details to those who wish to perform the operation. Chapter fifth—the last—explains the method of operating, containing accurate and beautiful illustrations of the parts concerned and the several steps in the operation, with drawings of instruments.

This book appears at a most opportune time, when so much thought, experiment, and inventive genius have been expended on the subject of the best methods of removing stone from the bladder, and revives interest in one of the oldest procedures and which probably has fallen unjustly into disrepute. It has no anatomical difficulties much, beyond avoiding the fold of peritoneum anterior to the bladder, is very easy to do, and must be thought of in certain cases where litho-lapaxy or lateral lithotomy would be full of danger.

